# Systematic Duffy phenotyping reveals high prevalence of Duffy-null genotype among neutropenic patients

**DOI:** 10.1515/almed-2026-0059

**Published:** 2026-07-09

**Authors:** Leire Saiz Sierra, Núria Ferrando Núñez, Maite Serrando Querol, Anna Marull Arnall

**Affiliations:** Laboratori Clínic Territorial Girona, Hospital Universitari Doctor Josep Trueta, Girona, Spain; Comisión de Hematología de la Sociedad Española de Medicina de Laboratorio (SEMEDLAB), Girona, Spain; Servei Bioquímica i Genètica Molecular, Centre de Diagnòstic Biomèdic (CDB), Hospital Clínic Barcelona, Barcelona, Spain

**Keywords:** Duffy-null phenotype, neutropenia; ACKR1, benign constitutional neutropenia

To the Editor,

Benign constitutional neutropenia (BCN) is characterised by persistently low absolute neutrophil counts (ANC) in the absence of immune dysfunction or increased infection risk. It is more prevalent in populations of African, Arab, and Afro-Caribbean ancestry and is caused by the single nucleotide polymorphism rs2814778 in the promoter region of the ACKR1 gene. This variant (the Duffy-null phenotype [Fy(a−b−)]) leads to the absence of Duffy antigen expression on erythrocytes, disrupting chemokine sequestration and leading to altered neutrophil trafficking and lower circulating counts. Despite its benign nature, BCN is frequently misidentified, resulting in unnecessary bone marrow biopsies, delayed treatments, and exclusion from clinical trials [1], [2], [3], [4].

This prospective case series includes 269 individuals with an ANC <1,500/µL, referred for Duffy phenotyping between January 2024 and April 2025. Patients were selected based on incidentally detected or routine neutropenia after an initial evaluation excluding evident secondary causes of neutropenia – including autoimmune disorders, thyroid disease, viral infections, hematologic malignancies, nutritional deficiencies, and drug-induced neutropenia. Referral for Duffy phenotyping was guided by the clinical suspicion of Duffy-null associated neutropenia in individuals with ancestry from populations in which the rs2814778 ACKR1 variant is prevalent, based mainly on self-reported country of origin and family background when available.

Phenotyping was performed via gel haemagglutination (IH-500, Bio-Rad). As a descriptive study, no formal control group was included.

Of the 269 patients, 233 (86.6 %) exhibited the Duffy-null phenotype, with a median ANC of 1,060/µL (IQR 930–1,240). The remaining 36 cases (13.4 %) presented non-null phenotypes: 16 Fy(a+b+) (5.9 %), 10 Fy(a+b−) (3.7 %), and 10 Fy(a−b+) (3.7 %). The remaining 36 cases (13.4 %) presented non-null phenotypes: 16 Fy(a+b+) (5.9 %), 10 Fy(a+b−) (3.7 %), and 10 Fy(a-b+) (3.7 %). Median ANC values ranged from 1,060 to 1,130/µL across Duffy phenotypes, without evident clinically relevant differences between Duffy-null and non–Duffy-null patients. In several non-Duffy-null cases, subsequent clinical evaluation identified secondary causes of neutropenia – including autoimmune disease, hypothyroidism, viral infections, and drug-induced suppression – despite no evident secondary cause at the time of referral for Duffy phenotyping [5].

**Figure 1:**
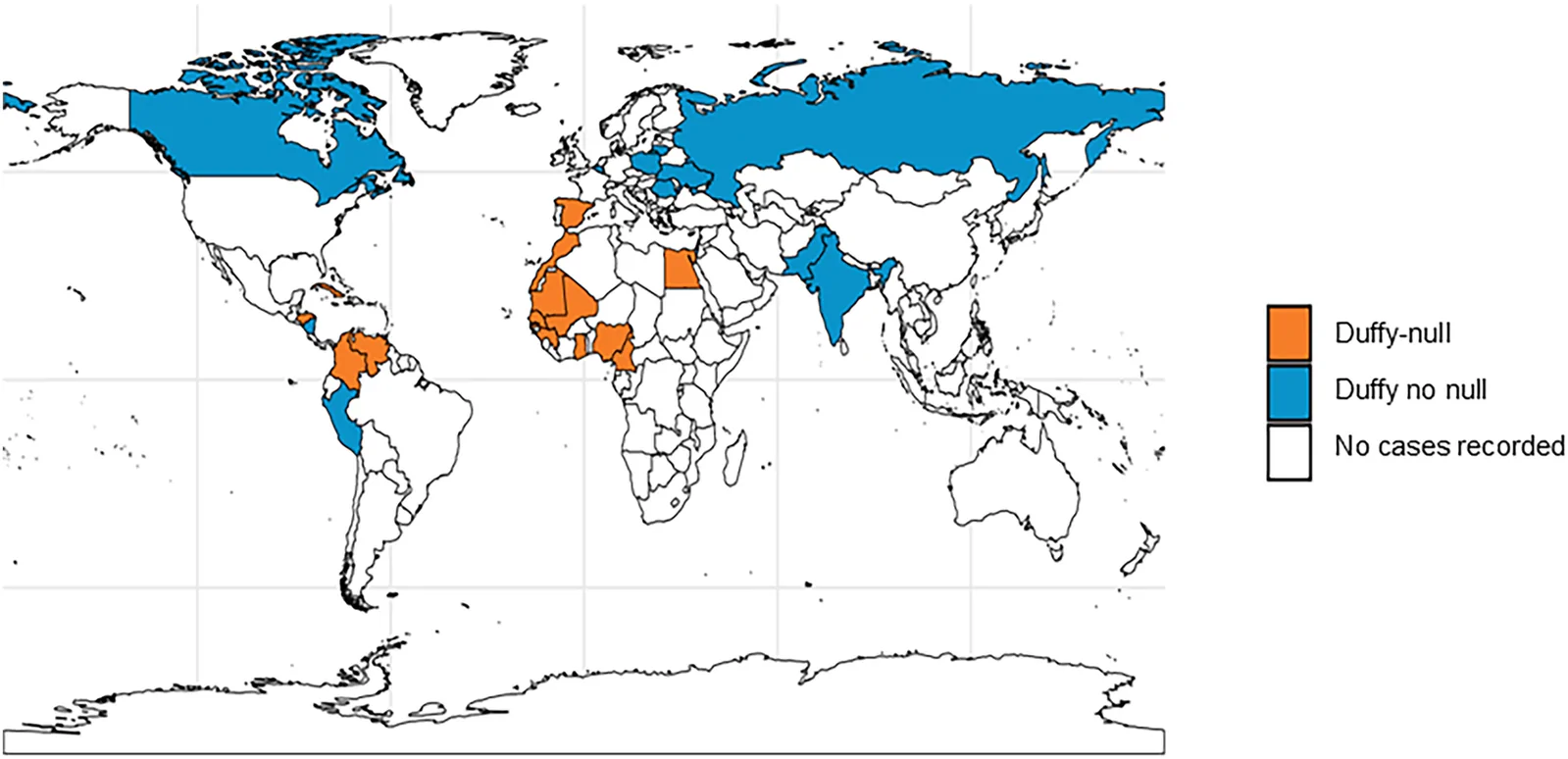
Geographic origin of patients in our cohort (n=269), colour-coded by Duffy phenotype: Orange=Duffy-null [Fy(a−b−)]; Blue=Duffy-non-null; White=no cases recorded.

As shown in Figure 1 and Table 1, the geographic origin of Duffy-null cases reflected the established global distribution of the rs2814778 variant. The geographic origin of Duffy-null cases reflected the established global distribution of the rs2814778 variant: the majority originated from Gambia (30.5 %), Morocco (16.3 %), and Senegal (16.3 %), followed by Guinea, Mauritania, Nigeria, and Mali. Importantly, 11.2 % of Duffy-null patients were born in Spain, possibly representing second-generation individuals of African descent, a clinically relevant subgroup who may not be readily identified by country of origin alone and in whom BCN may be overlooked. Smaller proportions originated from South America consistent with the transcontinental spread of this variant [2], 6].

**Table 1:** Geographic origin and Duffy phenotype of neutropenic patients (n=269).

Country of origin	Total cases n (%)	Duffy-null n (%)	Fy(a+b+) n (%)	Fy(a+b−) n (%)	Fy(a−b+) n (%)
Gambia	71 (26.4 %)	71 (30.5 %)	–	–	–
Morocco	46 (17.1 %)	38 (16.3 %)	4 (25.0 %)	2 (20.0 %)	2 (22.2 %)
Senegal	38 (14.1 %)	38 (16.3 %)	–	–	–
Spain	36 (13.4 %)	26 (11.2 %)	5 (31.2 %)	1 (10.0 %)	3 (33.3 %)
Guinea	10 (3.7 %)	10 (4.3 %)	–	–	–
Mauritania	10 (3.7 %)	10 (4.3 %)	–	–	–
Nigeria	10 (3.7 %)	10 (4.3 %)	–	–	–
Mali	9 (3.3 %)	9 (3.9 %)	–	–	–
Honduras	6 (2.2 %)	4 (1.7 %)	–	2 (20.0 %)	–
Colombia	3 (1.1 %)	3 (1.3 %)	–	–	–
Others^a^	30 (11.2 %)	14 (6.0 %)	7 (43.7 %)	5 (50.0 %)	4 (44.4 %)
Total	269 (100 %)	233 (86.6 %)	16 (5.9 %)	10 (3.7 %)	9 (3.3 %)

^a^‘Others’ includes countries with <3 cases each: Ghana, Ivory Coast, Venezuela, Cameroon, Cuba, Egypt, United States, Spain-Africa (Duffy-null); Poland, Ukraine, India, Russia, Romania (Fy[a+b+]); Peru, Pakistan, China (Fy[a+b−]); Algeria, Nicaragua (Fy[a−b+]).

This case series demonstrates that the Duffy-null phenotype accounts for the vast majority (86.6 %) of BCN cases in a multiethnic clinical setting in Spain, consistent with findings from other European and North American centres [7]. The comparable ANC values across Duffy phenotypes within the neutropenic cohort (medians 1,060–1,130/µL) suggest that non-Duffy-null cases require evaluation for secondary causes, whereas Duffy-null individuals with ANC in the BCN range (generally 1,000–1,500/µL) do not require further invasive workup [5].

The geographic distribution of Duffy-null cases reflects the established epidemiology of the rs2814778 variant, with predominance from West African countries where the allele frequency approaches 96 %. The identification of 11.2 % of Duffy-null patients born in Spain highlights an important clinical consideration [1], 2]: second-generation migrants may not be readily identified by country of origin alone, and reliance on birthplace could lead to missed diagnoses of BCN. This underscores the need for clinicians to consider ancestry rather than birthplace when evaluating unexplained neutropenia [6].

The clinical implications of this study are substantial. Recent evidence demonstrates that conventional ANC thresholds systematically exclude individuals with Duffy-null phenotype from cancer clinical trials – at commonly used thresholds of 1,500–2,000/µL, nearly 20–30 % of Duffy-null individuals would be excluded despite having no pathological neutropenia. Similarly, unnecessary invasive bone marrow examinations are performed in patients with unrecognised BCN, with one multicentre study finding that 63 % of African American patients undergoing bone marrow biopsy for isolated neutropenia had the rs2814778 genotype [3], 4], 8]. In psychiatric practice, undiagnosed BCN leads to inappropriate discontinuation of clozapine therapy, with genetic testing revealing that 24 % of patients on long-term clozapine had previously undiagnosed benign ethnic neutropenia [6].

Incorporating Duffy phenotyping into the diagnostic algorithm for unexplained neutropenia in at-risk populations represents a cost-effective strategy to prevent iatrogenic harm [5]. This is particularly relevant in European centres serving increasingly diverse populations, including second-generation migrants. Recent guidelines recommend that extensive evaluation should not be performed in asymptomatic, healthy patients with chronic mild neutropenia associated with the Duffy-null phenotype [5].

Clinical guidelines should adopt ethnic-specific ANC reference intervals for Duffy-null individuals. Recent multinational studies have established Duffy-null-specific reference ranges; implementation of these ranges would prevent misclassification of 21.7–50.9 % of Duffy-null individuals as neutropenic [6]. Furthermore, the terminology “benign ethnic neutropenia” should be replaced with “Duffy-null associated neutrophil count” to avoid perpetuating the notion that common phenotypes in non-White populations are abnormal [8].

In conclusion, this case series from a Spanish tertiary centre demonstrates the high prevalence of Duffy-null phenotype among patients referred for neutropenia evaluation and supports the integration of Duffy phenotyping into routine clinical practice to prevent misdiagnosis, unnecessary investigations, treatment delays, and unjustified exclusion from clinical trials.
